# Human iPSC-Derived Cardiomyocytes for Investigation of Disease Mechanisms and Therapeutic Strategies in Inherited Arrhythmia Syndromes: Strengths and Limitations

**DOI:** 10.1007/s10557-017-6735-0

**Published:** 2017-07-18

**Authors:** Simona Casini, Arie O. Verkerk, Carol Ann Remme

**Affiliations:** 1 Department of Clinical and Experimental Cardiology, Heart Center, Academic Medical Center, University of Amsterdam, Room K2-104-2, Meibergdreef 15, PO Box 22700, 1100 DE Amsterdam, The Netherlands; 2 Department of Medical Biology, Academic Medical Center, University of Amsterdam, Amsterdam, The Netherlands

**Keywords:** Induced pluripotent stem cells, Cardiomyocytes, Human, Arrhythmias, Pharmacology

## Abstract

During the last two decades, significant progress has been made in the identification of genetic defects underlying inherited arrhythmia syndromes, which has provided some clinical benefit through elucidation of gene-specific arrhythmia triggers and treatment. However, for most arrhythmia syndromes, clinical management is hindered by insufficient knowledge of the functional consequences of the mutation in question, the pro-arrhythmic mechanisms involved, and hence the most optimal treatment strategy. Moreover, disease expressivity and sensitivity to therapeutic interventions often varies between mutations and/or patients, underlining the need for more individualized strategies. The development of the induced pluripotent stem cell (iPSC) technology now provides the opportunity for generating iPSC-derived cardiomyocytes (CMs) from human material (hiPSC-CMs), enabling patient- and/or mutation-specific investigations. These hiPSC-CMs may furthermore be employed for identification and assessment of novel therapeutic strategies for arrhythmia syndromes. However, due to their relative immaturity, hiPSC-CMs also display a number of essential differences as compared to adult human CMs, and hence there are certain limitations in their use. We here review the electrophysiological characteristics of hiPSC-CMs, their use for investigating inherited arrhythmia syndromes, and their applicability for identification and assessment of (novel) anti-arrhythmic treatment strategies.

## Introduction

Life-threatening ventricular arrhythmias and sudden arrhythmic death typically occur in the setting of common cardiovascular pathologies associated with structural cardiac abnormalities, including myocardial ischemia/infarction, heart failure, and cardiomyopathy. In a subset of patients, often relatively young and otherwise healthy individuals, ventricular arrhythmias are the consequence of an inherited arrhythmia syndrome. Here, mutations in genes encoding ion channels, transporters, interacting proteins, or regulatory pathways lead to potentially pro-arrhythmic alterations of cardiac electrophysiological properties [1, 2]. Since correction of the genetic defect is as yet impossible, pharmacological treatment strategies are generally aimed at preventing or counteracting the detrimental functional effects of the mutation. In some cases, implantation of an implantable cardioverter defibrillator (ICD) is the only option for preventing sudden arrhythmic death, but ICD implantation may be associated with serious side effects and lower quality of life [3]. During the last two decades, significant progress has been made in the identification of genetic defects underlying inherited arrhythmia syndromes, which has provided some benefit through elucidation of gene-specific arrhythmia triggers and treatment, most notably for Long QT syndrome [1, 4]. However, for most arrhythmia syndromes, clinical management is hindered by insufficient knowledge of the functional consequences of the mutation in question, the pro-arrhythmic mechanisms involved, and hence the most optimal treatment strategy. Moreover, disease expressivity and sensitivity to therapeutic interventions often varies between mutations and/or patients, underlining the need for more individualized strategies.

Following identification of a putative mutation in a patient with an inherited arrhythmia syndrome, its functional consequences may be investigated in vitro. In the case of mutations in ion channel genes or known regulatory proteins, the impact on (dys)function of the ion channel in question is typically investigated through current measurements (using patch clamp analysis) in heterologous expression systems in which the mutated ion channel of interest is expressed (i.e., *Xenopus* oocytes, human embryonic kidney or HEK cells, and Chinese Hamster Ovary or CHO cells) [5]. This approach allows for elucidation of the effect of the mutation on current density, kinetics, and sensitivity to certain drugs. While certainly informative, these expression systems do not necessarily reflect the situation in the endogenous cardiomyocyte (CM) since they often lack accessory proteins and other cellular components required for proper ion channel function. Transgenic mice carrying specific mutations overcome many of these limitations. However, while recent progress (i.e., CRISPR-Cas9 technology) has significantly decreased both time and costs related to the generation of such mouse models, they are not suitable for high-throughput screening of rare inherited arrhythmia mutations. Mice display crucial differences in cardiac electrophysiological characteristics as compared to humans, including a high heart rate and a relatively short action potential secondary to differences in potassium currents [6, 7]. Moreover, transgenic mouse models lack the disease variability commonly observed in patients secondary to genetic background, comorbidity, and environmental factors. Hence, the electrophysiological consequences of mutations associated with inherited cardiac arrhythmias are ideally investigated in human CMs. However, the latter are not widely available since obtaining cardiac biopsies from patients is a highly invasive procedure and not without risk. Furthermore, it is difficult to keep human adult CMs in culture, limiting their applicability for more long-term studies.

The development of the induced pluripotent stem cell (iPSC) technology now provides the opportunity for generating and culturing iPSC-derived cardiomyocytes obtained from human material (human-induced pluripotent stem cell (hiPSC)-CMs) [8, 9]. These hiPSC-CMs enable investigation of patient- and/or mutation-specific disease mechanisms as well as identification and assessment of novel therapeutic strategies for arrhythmia syndromes. However, due to their relative immaturity, hiPSC-CMs also display a number of essential differences as compared to adult human CMs, and hence there are certain limitations in their use. The purpose of this review is to evaluate the applicability of hiPSC-CMs for investigation of inherited arrhythmia syndromes and assessment of (novel) anti-arrhythmic treatment strategies. To this end, we describe the electrophysiological characteristics of hiPSC-CMs, the various available tools for their functional analysis, and their strengths and limitations. In addition, we present an overview of pharmacological studies employing hiPSC-CMs models of inherited arrhythmia syndromes.

## General Characteristics of hiPSC-CMs

**Fig. 1:**
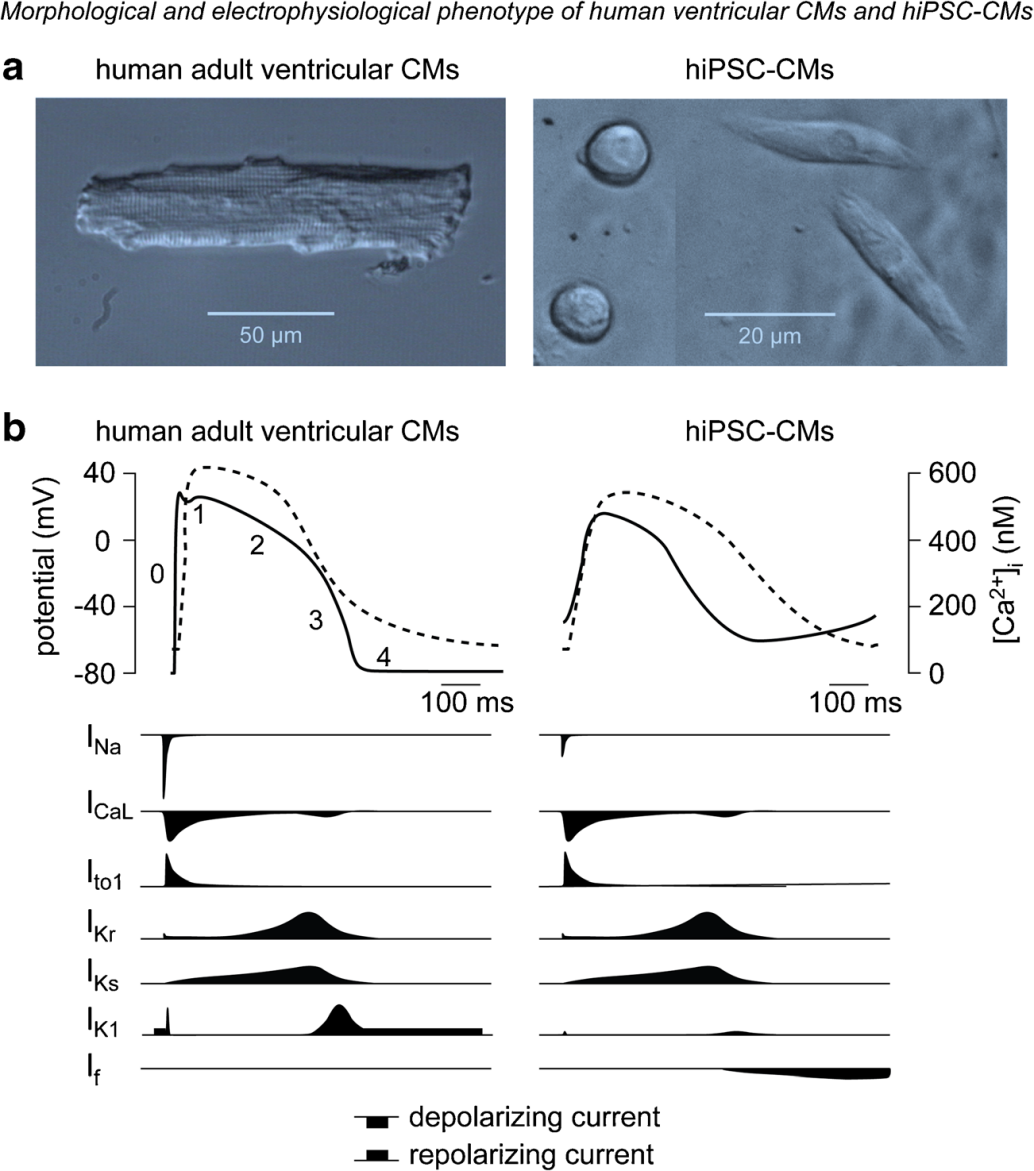
Morphological and electrophysiological phenotype of human-induced pluripotent stem cell-derived cardiomyocytes (hiPSC-CMs) and native human ventricular cardiomyocytes (CMs). **a** Morphological differences between human adult ventricular CMs and hiPSC-CMs; note the different scale. **b** Examples of action potential (*solid line*) and calcium transient (*dashed line*) in human adult ventricular CMs and hiPSC-CMs (*upper panels*), and schematic representation of the time course and magnitude of relevant ion currents (*lower panels*). Figure modified from [68] (**a**) and [15] (**b**)

Generation of hiPSCs starts by obtaining somatic cells from easily accessible human material, such as hair, blood, skin, fat, urine, or oral mucosa. These cells are reprogrammed to a pluripotent state by introducing pluripotency-associated genes, and hiPSCs thus generated can be kept in culture indefinitely. Next, hiPSCs are differentiated towards the cardiac lineage through a number of strategies (for review, see [10, 11]). After approximately 8–12 days, areas of beating cells typically appear, which can be microscopically dissected and dissociated into single CMs for subsequent electrophysiological and immunofluorescence analysis. This approach, which is both laborious and expensive, enables generation of patient-specific hiPSC-CMs for electrophysiological and pharmacological investigations. Today, hiPSC-CMs are also commercially available; these control lines can be used to introduce mutations using CRISPR-Cas9 technology followed by functional studies [12]. Conversely, isogenic lines can be created by repairing the mutation, thus serving as patient-specific controls [13, 14]. While they facilitate investigations in a human cardiomyocyte environment, an important limitation of hiPSC-CMs relates to the fact that they are immature and in fact share more similarities with fetal than with adult human CMs (for review, see [15, 16]). Typically, hiPSC-CMs express high levels of cardiac-specific genes (including Nkx2.5, cardiac troponin T, α-myosin heavy chain, α-actinin, myosin light chain 2, etc.) and display a striated pattern for α-actinin and myosin light chain, comparable to adult ventricular myocardium [17]. However, adult CMs are rod shaped and elongated, while hiPSC-CMs usually have a more round or multi-angular shape and are smaller in size (Fig. 1a). Moreover, in contrast to adult CMs, hiPSC-CMs display relatively disorganized sarcomeres and typically lack t-tubuli [18, 19]. The relative intrinsic immaturity of hiPSC-CMs is also reflected in important differences in functional characteristics as compared to adult CMs, including electrophysiology and excitation–contraction coupling. A number of approaches have been developed to enhance hiPSC-CMs maturity (see “Approaches for Improved Electrophysiological Measurements in hiPSC-CMs” section) leading to improved contractile, calcium handling, and electrophysiological properties [15, 16, 20, 21]. Nevertheless, the intrinsic properties of hiPSC-CMs need to be considered when employing these cells for investigation of arrhythmia disease mechanisms and assessment of (novel) therapeutic strategies.

## Electrophysiological Characteristics of hiPSC-CMs

### Action Potential

**Fig. 2:**
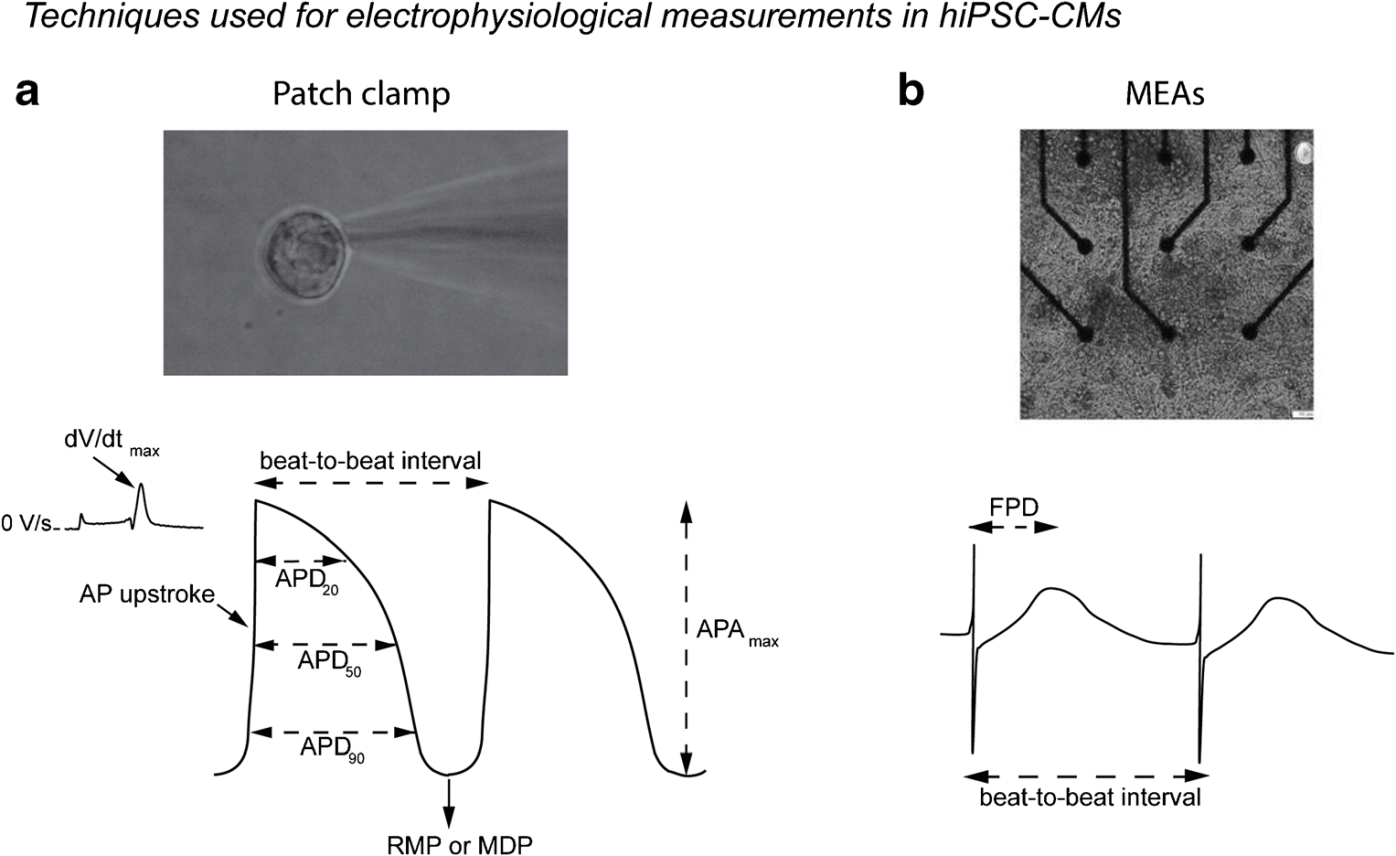
Examples of techniques used for electrophysiological measurements in hiPSC-CMs. **a** hiPSC-CMs patched with manual anpatch clamp technique (*upper panel*). Typical AP trace recorded in hiPSC-CMs with the use of the patch clamp technique (*lower panel*). **b** Cluster of hiPSC-CMs seeded on multi-electrode arrays (*MEAs*) (*upper panel*). Field potential trace obtained with MEAs (*lower panel*). Assessable parameters are indicated in the corresponding figures. *APD*_*20*_, *APD*_*50*_, and *APD*_*90*_ action potential duration at 20, 50, and 90% repolarization; *APA*_*max*_ maximal AP amplitude; *RMP* resting membrane potential; *MDP* maximal diastolic potential; *dV/dt*_*max*_ maximal upstroke velocity; *FPD* field potential duration. Upper panel reproduced from [122]

The cardiac action potential (AP) is divided in five different phases, i.e., phase 0 to 4 (Fig. 1b). The AP is initiated by a large, rapid influx of sodium (Na^+^) through Na^+^ channels, resulting in fast depolarization of the cell membrane, the so-called upstroke or phase 0 of the AP. Following the AP upstroke, there is a brief repolarizing phase (phase 1), resulting from efflux of potassium (K^+^) caused by activation of the transient outward K^+^ current (*I*_to1_). Next, inward flow of calcium (Ca^2+^) through L-type Ca^2+^ current (*I*_CaL_) leads to the plateau phase (phase 2). Finally, the membrane repolarizes to its original state due to activation of the rapid and slow delayed rectifier K^+^ channels (conducting the *I*_Kr_ and *I*_Ks_ currents, respectively) in phase 3 of the AP. Adult ventricular and atrial cardiomyocytes (but not nodal cells) also exhibit phase 4 in which the resting membrane potential (RMP) remains constant due to the presence of the inward rectifying K^+^ current *I*_K1_. With the use of the patch clamp technique, various AP parameters can be analyzed which reflect specific membrane current functions. These parameters include RMP or maximal diastolic potential (MDP), cycle length, AP amplitude, AP upstroke velocity, and AP duration (APD) at various levels of repolarization (i.e., APD_20_, APD_50_, and APD_90_) (Fig. 2a). For example, the RMP or MDP is an indication for steady-state K^+^ currents, such as *I*_K1_. AP upstroke velocity is a measure of sodium current (*I*_Na_) availability, while the various AP durations (APDs) reflect the various phases of repolarization consequent to the differential contribution of various membrane currents. APD_20_ is for example importantly regulated by *I*_CaL_ and *I*_to1_, while APD_90_ is importantly set by *I*_Ks_ and *I*_Kr_ (although some overlap exists).

The most common hiPSCs differentiation protocols predominantly generate cells classified as ventricular-like hiPSC-CMs [22, 23], although protocols aimed at specifically generating atrial-like [24] and nodal-like hiPSC-CMs [25] have also been recently described. The distinction between hiPSC-CMs cell type is often made based on AP phenotype, with a more negative MDP, a rapid AP upstroke, and a long plateau phase for ventricular-like APs. It is however questionable whether the subdivision in the various cell types based only on the AP shape is correct, since APs in hiPSC-CMs resemble a fetal-like phenotype. Single-cell mRNA expression measurements of hiPSC-CMs following patch clamp analysis have demonstrated a good correlation between expression of specific myocyte-lineage markers and designation of AP as ventricular-like, atrial-like, and nodal-like based on cellular electrophysiological features [26, 27]. However, caution should be taken when applying this selection method, since the cutoff values for the AP duration and upstroke velocities for the various cell types are arbitrary and differ among studies. The presence of specific currents may also be used to distinguish atrial and nodal CMs in the hiPSC-CMs population, such as the ultra-rapid delayed rectifier K^+^ current (*I*_Kur_) and the acetylcholine-sensitive K^+^ current (*I*_KACh_) for atrial cells [24] and the “funny current” (*I*_f_) and T-type Ca^2+^ current (*I*_CaT_) for nodal cells (see also “Ion currents” section) [25].

Comparison of hiPSC-CMs AP characteristics among various studies is difficult due to differences in experimental conditions used (variable recording temperatures, perforated vs. whole-cell configuration technique, spontaneous beating cells vs. triggered cells). Nevertheless, the most remarkable difference between hiPSC-CMs and native CMs relates to the observation that in most studies, hiPSC-CMs (including ventricular-like and atrial-like cells) by themselves are spontaneously active, with beating rates of approximately 30 to 120 beats per minutes (for review, see [28, 29]). Moreover, APs recorded in ventricular-like hiPSC-CMs present intrinsically less negative RMP/MDP, lower upstroke velocity, and a less prominent notch (phase 1) as compared to freshly isolated human ventricular adult CMs [28, 29] (Fig. 1b). These characteristics reflect the relative immature state of hiPSC-CMs and constitute potential limitations for their use as adequate models of arrhythmia syndromes. However, certain technical approaches and experimental protocols may be employed to overcome these limitations (see “Approaches for Improved Electrophysiological Measurements in hiPSC-CMs” section).

### Ion Currents

Because of the clear differences in AP shape between native, adult CMs, and hiPSC-CMs, it is likely that differences exist in the functional availability of the various cardiac ion channels due to differences in density and gating properties. Thus, before establishing hiPSC-CMs as a cellular model for investigating cardiac arrhythmia syndromes, it is important to carry out a detailed comparison between ion currents in hiPSC-CMs with those in native CMs (Fig. 1b). Such a comparison remains difficult, since only few studies have investigated in detail ion current characteristics in healthy, adult human ventricular CMs. Furthermore, ionic current properties are dependent on experimental conditions and protocols, as well as tissue heterogeneity (transmural, atrial vs. ventricle vs. nodal). Moreover, it should be noted that ion channel function is tightly regulated by accessory subunits, interacting proteins, and intracellular metabolism. Knowledge of these regulatory factors and processes in hiPSC-CMs is still rudimentary, but they are likely not identical to those in the adult (human) ventricular CMs. In the sections below, an overview is presented of the characteristics of the major ion currents in hiPSC-CMs (for details, see Table 1). Table 2 lists the most relevant inhibitors typically used to study the various currents. Since mostly ventricular-type hiPSC-CMs are obtained with the currently used differentiation protocols, a comparison is made between hiPSC-CMs and healthy native human ventricular CMs, unless stated otherwise.

**Table 1:** Biophysical properties of ion currents in hiPSC-CMs and healthy human ventricular myocytes

	Cell type	Current density (pA/pF)	Refs	*V*_1/2_ of activation (mV)	Refs	*V*_1/2_ of inactivation (mV)	Refs	Recovery from inactivation (ms)	Refs
*I*_Na_	hiPSC-CMs	From −12.2^a^ to −272.2	[30–37, 108]	From −25.5 to −42.4	[30, 32, 33, 35, 37, 40]	From −61.4 to −85.0	[30, 32, 33, 35, 37, 40]	*τ* = 60.4 *t*_1/2_ = 10.8 *τ*_f_ = 6.4, 6.0 *τ*_s_ = 103.6, 118.1	[35, 37, 40, 108]
	Human VM	−49.0	[38]	−51.0	[38]	−102.0	[38]	NA	NA
*I*_CaL_	hiPSC-CMs	From −6.6 to ~−58	[14, 30, 33, 34, 44, 45]	From ~−12 to ~−28	[14, 30, 33]	−29.1, ~−45	[30, 33]	NA	NA
	Human VM	−10.2, −3.8	[46, 47]	−4.7, −4.2	[46, 47]	−19.3, −23.5	[46, 47]	NA	NA
*I*_to1_	hiPSC-CMs	From ~1.3 to ~19	[26, 30, 33, 52]	From ~10 to ~20	[26, 30, 33, 52]	~−40, −41.1	[33, 52]	*τ*_f_ = 200 *τ*_s_ = 2380	[52]
	Human sub-epi VM	6.8, 10.6	[49, 50]	9.7, 29.1	[49, 50]	−31.9, −9.5	[49, 50]	*τ* = 24 *τ*_s_ = 9621 *τ*_f_ = 46.1	[49, 50]
	Human sub-endo VM	4.4, 2.6	[49, 50]	23.1, 32.0	[49, 50]	−25.3, −17.6	[49, 50]	*τ*_f_ = 25.0, 90.6 *τ*_s_ = 328.0, 3109.0	[49, 50]
	Human VM	9.3, 8.5^b^	[46, 51]	~30, ~20	[46, 51]	−27.3	[51]	*τ* = 27.8	[51]
*I*_Kr_	hiPSC-CMs	From ~0.18 to ~2.5	[13, 14, 26, 30, 34, 40, 53–56]	From ~−10 to ~−36	[13, 14, 30, 34, 40, 53, 56]	NA		NA	
	Human VM	0.31, ~0.25, ~0.6	[46, 57, 58]	~+22, −5.74	[46, 57]	NA		NA	
*I*_Ks_	hiPSC-CMs	From 0.22 to ~2.9	[12, 14, 26, 30, 34, 61, 62]	From ~−10 to ~+22	[12, 14, 26, 30, 61, 62]	X		X	
	Human VM	0.18	[60]	NA		X		X	
*I*_K1_	hiPSC-CMs	From ~−0.8 to −5.1	[30, 34, 53, 68]	X		X		X	
	Human VM	From −3.6 to −32.1	[68]	X		X		X	
*I*_f_	hiPSC-CMs	−4.1, −0.9, ~−3.0	[30, 34, 48]	−84.6	[30]	X		X	
	Human VM	−1.18	[71]	−110, −80.0	[71, 72]	X		X	

*I*_*Kr*_ and *I*_*Ks*_ density measurements refer to maximal peak tail current, except for the studies of Sala et al. [14], Terrenoire et al. [40], and Ma et al. [30] where *I*_Kr_ and *I*_Ks_ are measured at the end of the depolarizing step;

*VM* ventricular myocytes; *V*_*1/2*_ (in)activation, half-voltage of (in)activation; *τ*_*f*_ and *τ*_*s*_ fast and slow time constants of recovery from inactivation, respectively; *τ* time constant of recovery from inactivation (curve fitted with a mono-exponential equation); *t*_*1/2*_ time for half of the channels to recover from inactivation; in hiPSC-CMs, *I*_to1_ density is measured at +40 mV, while in human VM is measured between +40 and +60 mV; *I*_*k1*_ density values are measured at −100 mV; *X* channel property does not exist; *NA* not assessed or measured; ~estimated from figure; *sub-epi* subepicardial myocytes; *sub-endo* subendocardial myocytes

^a^ *I*_Na_ recorded with low extracellular sodium (7 mM Na^+^) solution [108]

^b^Average *I*_to1_ density between 0 and +60 mV [51]

**Table 2:** Blockers commonly used to investigate specific ion currents

Ion current	Blockers
Sodium current (*I*_Na_)	Flecainide, TTX
L-type calcium current (*I*_CaL_)	Nifedipine
T-type calcium current (*I*_CaT_)	Nickel
Late sodium current (*I*_NaL_)	GS967, ranolazine
Transient outward potassium current (*I*_to1_)	4-Aminopyridine
Rapid delayed rectifier potassium current (*I*_Kr_)	E-4031
Slow delayed rectifier potassium current (*I*_Ks_)	JNJ303, chromanol 293B
Inward rectifier potassium current (*I*_K1_)	Barium
“Funny” current (*I*_f_)	Ivabradine

*TTX* tetrodotoxin

#### Sodium Current (*I*_Na_)

The cardiac sodium channel Na_v_1.5, encoded by the *SCN5A* gene, carries the inward *I*_Na_ which is responsible for the rapid upstroke of the AP in CMs and consequently for proper cardiac excitability and impulse propagation. In the majority of studies, maximal peak *I*_Na_ amplitudes appear considerably larger in hiPSC-CMs [30–37] than those reported for native human ventricular CMs [38], whereas *SCN5A*/Na_v_1.5 expression levels have been reported to be lower in hiPSC-CMs as compared to adult CMs [39]. Hence, the apparent larger *I*_Na_ density in hiPSC-CMs as compared to native CMs is likely due to experimental conditions (i.e., reduced extracellular Na^+^ concentration used in adult human ventricular CMs studies) rather than an increased number of functional channels. Conversely, the low upstroke velocity (a measure of Na^+^ channel availability) often observed in hiPSC-CMs does not necessarily reflect low *I*_Na_ density but is likely due to the fact that at more positive MDP, a proportion of Na^+^ channels is inactivated and therefore not functionally available. Values for half-voltage dependence of (in)activation (V_1/2_), the voltage at which half channels are (in)activated, were found to be similar among the several control hiPSC-CMs lines analyzed in different studies (Table 1) [30, 32, 33, 35, 37, 40]. However, *V*_1/2_ values of (in)activation are generally shifted towards more positive potentials in hiPSC-CMs as compared to the values reported for ventricular CMs isolated from healthy individuals [38] (Table 1). Similar to human adult CMs, Na^+^ channels in hiPSC-CMs are sensitive to micromolar doses of the sodium channel blocker tetrodotoxin [30, 41] (Table 2). Thus, *I*_Na_ characteristics appear comparable between hiPSC-CMs and human adult CMs. Although the initial large inward *I*_Na_ that contributes to the AP upstroke is for the most part rapidly inactivated, a small fraction of the current (designated the late sodium current, *I*_NaL_) persists throughout the duration of the AP plateau. Enhanced *I*_NaL_ is typically found during heart failure and long QT syndrome type 3 (LQT3, see “Pharmacological Studies for Inherited Arrhythmia Syndromes Using hiPSC-CMs” section) [42]. *I*_NaL_ in freshly isolated healthy human CMs is small [38]; in some hiPSC-CMs studies, *I*_NaL_ was observed to be similarly small [31, 35], while in other studies, it was reported to be absent [37, 40].

#### Calcium Current (*I*_Ca_)

Two types of Ca^2+^ channels are present in the human heart, the L-type and T-type channels, i.e., *I*_CaL_ and *I*_CaT_. For hiPSC-CMs, the presence of *I*_CaT_ has been debated: *I*_CaT_ was reported in a subset of hiPSC-CMs in one study [41], while in another, the current was not detected [30]. Similarly, *I*_CaT_ is not functionally present in healthy human native ventricular CMs but is present in the human heart conduction system, where it plays a role in facilitation of pacemaker depolarization [43]. *I*_CaL_ plays a crucial role in maintaining the plateau phase of the AP and in excitation–contraction coupling in cardiac cells. Several studies have shown the presence of a robust *I*_CaL_ in hiPSC-CMs [14, 30, 33, 34, 44, 45]. With the exception of the study of Veerman et al., [33], *I*_CaL_ densities [14, 30, 34, 44, 45] and mid-voltage inactivation values in hiPSC-CMs [30] are similar to those described for adult ventricular CMs [46, 47]. Voltage dependence of activation is generally shifted towards more negative potentials in hiPSC-CMs as compared to human native CMs (Table 1), while time course of current inactivation, analyzed in only one study [45], was similar to that reported in human ventricular CMs [46, 47]. The L-type Ca^2+^ channel blocker nifedipine decreased *I*_CaL_ [30, 41, 48] and resulted in shortening of the AP in hiPSC-CMs with minimal effects on upstroke velocity [30, 34]. Thus, hiPSC-CMs have robust *I*_CaL_ with characteristics close to that observed in adult human CMs.

#### Transient Outward Potassium Current (*I*_to1_)

Efflux of K^+^ secondary to activation of the transient outward potassium current (*I*_to1_) results in the relatively short-lasting repolarization phase 1 of the AP. A wide variation in peak current densities and kinetics has been reported for *I*_to1_ in both native ventricular CMs [46, 49–51] and hiPSC-CMs [26, 30, 33, 52] (Table 1), which can in part be explained by differences in experimental conditions. In addition, marked regional differences in density and kinetic properties of *I*_to1_ exist between subendocardial and subepicardial layers of human ventricular myocardium, contributing to the transmural electric gradient. In particular, subepicardial myocytes display increased *I*_to1_ density and faster recovery as compared to subendocardial CMs [49, 50]. Only one study analyzed *I*_to1_ recovery from inactivation in hiPSC-CMs, which proved to be markedly slower than previously reported in human adult epicardial ventricular CMs but comparable to values reported for human adult endocardial CMs [52]. In hiPSC-CMs with a relatively depolarized MDP, the functional relevance of *I*_to1_ may be limited, since a large proportion of the channels will be inactivated under these conditions. This, in combination with the slow recovery of the channel, which would further reduce *I*_to1_ availability, likely explains the less pronounced phase 1 of the AP in hiPSC-CMs [52].

#### Slow and Rapid Delayed Rectifier Potassium Currents (*I*_Ks_ and *I*_Kr_)

The rapid component of the delayed rectifier potassium current (*I*_Kr_) has been reported in hiPSC-CMs [13, 14, 26, 30, 34, 40, 53–56], with most studies showing a maximal density similar to or higher than that observed in native human ventricular CMs [46, 57, 58] (Table 1). In line with this, blockade of *I*_Kr_ by E-4031 (Table 2) in hiPSC-CMs resulted in a significant AP prolongation and induced early afterdepolarizations (EADs) [30, 34, 54, 55, 59]. Thus, *I*_Kr_ plays a prominent role in the repolarization phase of hiPSC-CMs AP. Moreover, mainly due to absence or low expression of *I*_K1_, *I*_Kr_ plays an important role in the determination of the MDP in hiPSC-CMs [53]. In native human ventricular CMs, the slow component of the delayed rectifier potassium current (*I*_Ks_) was reported to be present in approximately half of the cells studied, with a maximal current density of 0.18 pA/pF [60], a value similar to what was reported by Egashira et al. (~0.22 pA/pF) [61] and Ma et al. (0.31 pA/pF) [30] in hiPSC-CMs. In contrast, other studies showed higher values for *I*_Ks_ density in hiPSC-CMs [12, 14, 26, 34, 62] (Table 1). This variability in current density may be partly explained by altered expression of the β-subunit KCNE1, as suggested by a study in human embryonic stem cell-derived CMs (hESC-CMs) [63].

Studies on the functional relevance of *I*_Ks_ have demonstrated mixed results. In dog or non-diseased human ventricular CMs, *I*_Ks_ blockade with chromanol 293B, HMR-1556, and L-735,821 did not significantly prolong APD at baseline but only in the presence of β-adrenergic stimulation (enhancing *I*_Ks_ and accelerating its activation) or in the setting of decreased repolarization reserve (i.e., *I*_Kr_ blockade) [64, 65]. The conclusion that *I*_Ks_ does not play a role in repolarization under resting conditions has however been challenged by Towart et al. [66] who found that JNJ303, an *I*_Ks_ inhibitor with enhanced potency, evoked torsades de pointes arrhythmias in anesthetized dogs [66]. In hiPSC-CMs, Ma et al. showed that *I*_Ks_ blockade by chromanol 293B resulted only in minimal prolongation of the AP [30]; on the other hand, it significantly prolonged field potential duration (reflecting the QT interval; see also “Multi-Electrode Arrays” section) in a multicellular hiPSC-CMs preparation in another study [61]. Upon β-adrenergic stimulation with isoprotenerol, *I*_Ks_ amplitude was increased in control hiPSC-CMs, and AP duration was reduced accordingly [26]. In contrast, in the study of Zhang et al., AP duration was unaffected in control hiPSC-CMs after application of noradrenaline [12]. Of note, patients carrying heterozygous or homozygous mutations in the *KCNQ1* gene encoding *I*_Ks_ show markedly prolonged QT intervals under resting conditions (see also “Long QT Syndrome Type 1” section). Importantly, hiPSC-CMs from these patients display significant AP prolongation [12, 26], suggesting a functional role for *I*_Ks_ in repolarization control in hiPSC-CMs.

#### Inward Rectifier Potassium Current (*I*_K1_)

The inward rectifier K^+^ current (*I*_K1_) is important for maintaining the RMP in atrial and ventricular CMs [67]. In adult human CMs, *I*_K1_ density varies between 3.6 and 32.1 pA/pF, depending on the different experimental conditions [68]. *I*_K1_ density in hiPSC-CMs [30, 34, 53, 68] is considerably smaller, and *KCNJ2* mRNA expression (encoding the α-subunit of the channel) is correspondingly low [39]. The small *I*_K1_ density in hiPSC-CMs likely contributes to the frequently observed spontaneous activity in these cells. Furthermore, the consequent depolarized RMP of hiPSC-CMs also significantly affects other AP characteristics, including upstroke velocity and duration. To overcome this limitation, various studies have artificially introduced *I*_K1_ into hiPSC-CMs, either by in silico injection or viral transfection (see “Approaches for Improved Electrophysiological Measurements in hiPSC-CMs” section).

#### “Funny” Current (*I*_f_)

*I*_f_ is an inward, depolarizing current activating at hyperpolarized membrane potentials. It is mediated by the family of the HCN channels (HCN1–4) with *HCN4* being the most abundant isoform underlying *I*_f_ in sinoatrial cells [69]. The presence of *I*_f_ has also been demonstrated in human atrial CMs [70] and in human ventricular CMs [71, 72]. A relatively large *I*_f_ density has been reported in hiPSC-CMs [30, 34, 48], which may be attributed to the fact that hiPSC-CMs express higher levels of the *HCN4* isoform as compared to adult human ventricular CMs [39]. In the study of Ma et al., *I*_f_ in hiPSC-CMs was reported to be activated already at relatively depolarized membrane potentials (negative to −60 mV), suggesting that it may contribute to the spontaneous activity often observed in these cells [30]. However, this observation was not confirmed in a recent paper of Zhang and colleagues [48] where *I*_f_ activated at potentials negative to −80 mV; moreover, these authors demonstrated that application of ivabradine, a selective *I*_f_ blocker, had no effect on the spontaneous beating frequency in hiPSC-CMs.

### Intracellular Ca^2+^ and Na^+^ Homeostasis

The intracellular Ca^2+^ (Ca^2+^_i_) transient which underlies the contraction of a myocyte is triggered by Ca^2+^ influx through *I*_CaL_, which results in release of Ca^2+^ from the sarcoplasmic reticulum (SR) via ryanodine-2 (RyR2) channels (i.e., Ca^2+^-induced Ca^2+^ release). The subsequent decline of Ca^2+^_i_ (required for diastolic relaxation) occurs mainly through reuptake into the SR mediated by sarco/endoplasmic reticulum Ca^2+^-ATPase (SERCA) and through extrusion of Ca^2+^ via the Na^+^-Ca^2+^ exchanger (NCX) in the plasma membrane [73]. Diastolic Ca^2+^_i_ is determined by this Ca^2+^_i_ decline and by the magnitude of “leak” of Ca^2+^ from the SR through RyR2 channels [74]. An additional mechanism for increased diastolic Ca^2+^_i_ is consequent to elevation of intracellular Na^+^ (Na^+^_i_) levels; the latter is controlled by a fine balance between Na^+^ influx (through Na^+^ channels, NCX, and Na^+^/H^+^ exchanger) and Na^+^ extrusion (via Na^+^/K^+^-ATPase) [75]. An increase of Na^+^_i_ will result in intracellular Ca^2+^ overload via enhanced reverse-mode NCX activity [76]. While Ca^2+^ is essential for cardiac contraction, increased diastolic Ca^2+^_i_ and/or spontaneous Ca^2+^ release from the SR may set the stage for cardiac arrhythmias. Thus, Ca^2+^_i_ and Na^+^_i_ are tightly regulated within CMs, and their dysregulation may have profound pro-arrhythmic consequences.

So far, only a few studies have investigated Ca^2+^_i_ and Na^+^_i_ in hiPSC-CMs. Key Ca^2+^ handling proteins are expressed in hiPSC-CMs, and the presence of functional SR and RyR activity has been demonstrated [44, 45, 77–79]. Due to limited access to freshly isolated healthy human ventricular CMs, Hwang et al. compared hiPSC-CMs with rabbit and mouse CMs [44]. They found that Ca^2+^_i_ transients in hiPSC-CMs are largely similar to those in rabbit and mouse CMs, except for a much slower Ca^2+^_i_ transient rise and decay in hiPSC-CMs [44]. The slower rise is likely the result of a poor coupling between Ca^2+^ influx through *I*_CaL_ and SR Ca^2+^ release through RyRs due to lack of t-tubuli in hiPSC-CMs. The slower Ca^2+^_i_ transient decay may be related to the immature state of hiPSC-CMs. Indeed, Ca^2+^_i_ transient decay became faster by prolongation of the time after induction of cardiac differentiation [44]. The relative contribution of the Ca^2+^_i_ removal pathways, including SERCA and NCX, was not significantly different from adult rabbit CMs [44]. Thus, these findings suggest both similarities and dissimilarities in Ca^2+^-handling between hiPSC-CMs and adult CMs.

## Electrophysiological Studies Using hiPSC-CMs: Technical Considerations

A variety of invasive and non-invasive methods exists for the electrophysiological analysis of hiPSC-CMs, including patch clamp methodology and sharp electrode measurements, multi-electrode arrays (MEAs), and voltage-sensitive fluorescence (Fig. 2). Each technique has specific strengths and limitations in hiPSC-CMs research as discussed in more detail below.

### Patch Clamp Technique

The patch clamp technique is relatively labor intensive and requires skilled and experienced operators. Yet, it is considered the gold standard for electrophysiological research since it is the most informative method, allowing both the recording of membrane currents and AP parameters (Figs. 1b and 2a) [80]. It entails gently pressing a relatively blunt glass pipette (2–4 MΩ) against a cell membrane after which suction is applied to obtain a high resistance, omega-shaped, seal. After gaining access to the cell, the “whole-cell patch clamp configuration” is obtained which allows for measurement of APs and membrane currents in “current” and “voltage” clamp, respectively. Patch clamp technique can be performed manually (positioning the pipettes with the help of micro-manipulators) or with the so-called automated patch clamp technique [81–83] (see “Automated Patch Clamp” section).

#### Current Clamp

In the current clamp mode, the current injected through the patch pipette is controlled while the free-running membrane potential of the cell is recorded. Current clamp allows measurements of APs that may either occur spontaneously or in response to an injected stimulus current via the patch pipette. A population of hiPSC-CMs typically contains both spontaneous beating and quiescent cells. In most studies, spontaneously beating hiPSC-CMs are selected to measure APs, since beating cells are considered to be CMs. However, spontaneously beating hiPSC-CMs are frequently depolarized and have a diastolic (phase 4) depolarization phase, which further depolarizes the membrane potential to around −40 mV prior to AP onset. Such a large depolarization inactivates various membrane currents such as *I*_Na_, and *I*_to1_, and increases the importance of *I*_Ks_ and *I*_Kr_ in setting the diastolic membrane potential [84]. To partially overcome these limitations, in some studies quiescent hiPSC-CMs able to contract on field stimulation are specifically selected for analysis [31]. However, selecting cells this way is technically challenging and time consuming.

To obtain whole-cell configuration, both the ruptured or perforated “whole-cell” patch clamp methodology may be used. Although access to the cell is typically better in ruptured patch, Ca^2+^-buffers (such as EGTA) are often added to the pipette solution when using this technique, which may modulate intracellular Ca^2+^ cycling and consequently affect cardiac contraction, AP morphology, and automaticity in hiPSC-CMs via Ca^2+^-sensitive ion channels and exchangers [85]. This is less of an issue when employing the perforated patch technique, which also provides more stable AP waveforms over time. In hiPSC-CMs studies, the current clamp technique can be used to measure APs from single cells as well as from clusters [86]. However, hiPSC-CMs clusters may contain a mixed population of “atrial-like,” “ventricular-like,” and “nodal-like” CMs, as well as cells which are not cardiomyocytes, and coupling between hiPSC-CMs and the latter may affect the AP depolarization and repolarization phase. These considerations need to be taken into account.

#### Voltage Clamp

In voltage clamp mode, the membrane potential is held at a set voltage level through a feedback circuit patch clamp amplifier, which allows the recording of the net membrane current at a given membrane potential. Applying dedicated voltage clamp protocols, ion current densities (defined as current divided by cell size) and various physiological properties, i.e., voltage dependency of (in)activation, recovery from inactivation, slow inactivation, and speed of current (in)activation and deactivation can be studied in single cells under carefully controlled conditions (see also “Electrophysiological Characteristics of hiPSC-CMs” section).

#### Automated Patch Clamp

While the manual patch clamp technique (as described in “Current Clamp” and “Voltage Clamp” sections) is regarded as the gold standard for electrophysiological studies, experimental procedures can be complex and time consuming, resulting in low throughput. In contrast, automated patch, by allowing multiple recordings in parallel, can increase data throughput 10- to 100-fold depending on the ion channel under investigation and the platform used [16]. With automated patch clamp, the traditional glass pipette is replaced by an aperture at the bottom of a well through which an applied negative pressure patches the cell membrane. While this allows the automatization of the patch clamp process, this automated technique requires high quality, high density, and homogenous single-cell suspensions [81–83], which can be challenging since hiPSC-CMs are relatively expensive to produce on a large scale and highly sensitive to enzymatic dissociation into single cells. Moreover, the automated patch technique does not allow for selection of cells to be measured, i.e., hiPSC-derived cardiomyocytes vs. fibroblasts, or hiPSC-CMs labeled with GFP following viral transfection or other genetic approaches. Ma et al. compared manual and automated patch in hiPSC-CMs and reported a variable success rates for planar automated patch clamp analysis, with acceptable recordings obtained in ~50% of measurements; however, they observed differences in current density and cell capacitance between automated and manually patched cells [30]. Some of these observed differences may be consequent to the hiPSC-CMs dissociation protocols used for manual vs. automated patch clamp experiments; for the latter, trypsin is often used which may impact on recording quality by affecting membrane proteins [87]. Using an alternative two-step dissociation protocol (consisting of trypsin dissociation, reseeding at a low density, and reharvesting by gentle dissociation with accutase), Rajamohan and colleagues were able to increase the number of successful automated recordings [88]. Thus, electrophysiological analysis of hiPSC-CMs using high-throughput automated patch approach is feasible [30, 88], but certain limitations need to be considered.

### Sharp Microelectrode Measurements

The intracellular sharp microelectrode technique is a traditional and well-established tool to measure accurate voltage or electrical currents passing through the membrane. In contrast to the patch clamp technique, cells or tissue are impaled with one or two sharp glass microelectrodes (>30 MΩ), which are filled with a rather non-physiological, high K^+^ solution. Two electrodes are needed to perform voltage clamp experiments, but one electrode is enough to perform current clamp measurements. The insertion of the microelectrodes into cells and/or tissue may result in some damage and consequent membrane depolarization; smaller cells (i.e., hiPSC-CMs) are more sensitive to this potential limitation. The sharp electrode technique is often used to record APs from hiPSC-CMs clusters and hence is associated with the potential limitation of mixed cell types within these clusters.

### Multi-Electrode Arrays

While the intracellular patch clamp and sharp microelectrode methodology generates high-quality AP and membrane current data, it is considered laborious and time consuming with multiple technical issues potentially preventing successful recordings. In addition to automated patch, non-invasive measurements of extracellular signals with MEAs are increasingly used in hiPSC-CMs research. From the electrical signals (field potentials, FPs), beating frequency and field potential duration (FPD), considered to resemble QT interval and AP duration, can be determined (Fig. 2b). MEAs allow long-term measurements of FPs from clusters and monolayers of hiPSC-CMs and are often used to test (new) compounds or genetic defects, particularly in relation to their effect on AP repolarization and QT interval [59, 89]. MEA measurements are often performed in spontaneous beating clusters and monolayers and few MEA setups have implemented methods for electrical stimulation. Thus, the majority of measurements are performed on multicellular tissue which is not paced at a fixed frequency, and hence compounds which alter cycle length may consequently affect field potential characteristics indirectly. To normalize for differences in beating rates, QT intervals are typically corrected using the Bazett or Fridericia formula. While this type of correction is efficient in patients, it may not be accurate in such in vitro conditions [14]*.* Moreover, in MEA experiments, it is difficult to distinguish between direct effects of compounds and genetic defects on FPDs or indirect effects through alterations of other AP parameters such as MDP, since information on the latter is not easily obtained using this technique [90]. In addition, the various cell types and coupling between CMs in multicellular preparations may dilute precise determination of effects. Moreover, hiPSC-CMs measured with MEAs are less sensitive to compounds than single cells [59], but this is a general finding in multicellular preparations. Finally, MEAs do not allow for assessment of biophysical parameters of specific ion currents, a prerequisite when assessing potential (novel) therapeutic approaches for inherited arrhythmic disorders. On the other hand, local activation times can be determined at each electrode within the MEA, allowing for generation of detailed activation maps and conduction velocity measurements. Conduction velocity in hiPSC-CMs monolayers is, however, relatively slow (10–20 cm/s compared to 60 cm/s in adult human left ventricle) [91, 92], likely due to differences in *I*_Na_ distribution and availability, connexin distribution, and morphology and geometry of hiPSC-CMs (see [15]).

### Fluorescence Measurements

Another non-invasive method to assess electrical activity in a high-throughput manner is through voltage-sensitive fluorescence. For this, hiPSC-CMs can be loaded for instance with the voltage-sensitive dye di-4-ANEPPS [93]. Voltage fluorescence can be used to quantify conduction velocities in multicellular preparations, as well as AP parameters in isolated hiPSC-CMs or clusters. For multicellular preparations and clusters, the potential associated disadvantages of effects of coupling, changes in MDP, and spontaneous activity mentioned above should be taken into account. In addition, motion artifacts need to be prevented to obtain stable signals, and therefore an inhibitor of myosin-actin interaction is often used, such as 2,3-butanedione monoxime (BDM). Both BDM and the voltage-sensitive dye, however, may have impact on basic electrical properties. In hiPSC-CMs, calcium handling properties can additionally be studied using calcium-sensitive dyes such as Fluo-2AM or Indo-1AM [44, 79]. However, fluorescent dyes can be phototoxic and are therefore not suitable for prolonged recordings. Alternatively, genetically engineered hiPSC-CMs expressing a voltage (ArcLight) fluorescent indicator can be used, which has the advantage that specific hiPSC-CMs cell types can specifically be measured by use of specific promotors for over-expression [94]. While fluorescent approaches may be implemented for initial high-throughput assessment of compounds (screening for novel drugs and/or cardiac safety assays) [95], they do not provide information on ion current characteristics. Furthermore, the lack of reference values (i.e., 0 mV level) is an important drawback since small variations in MDP may not be detected, yet may still importantly impact on ion channel availability and AP characteristics as recently discussed in more detail by others [96–98].

### Approaches for Improved Electrophysiological Measurements in hiPSC-CMs

**Fig. 3:**
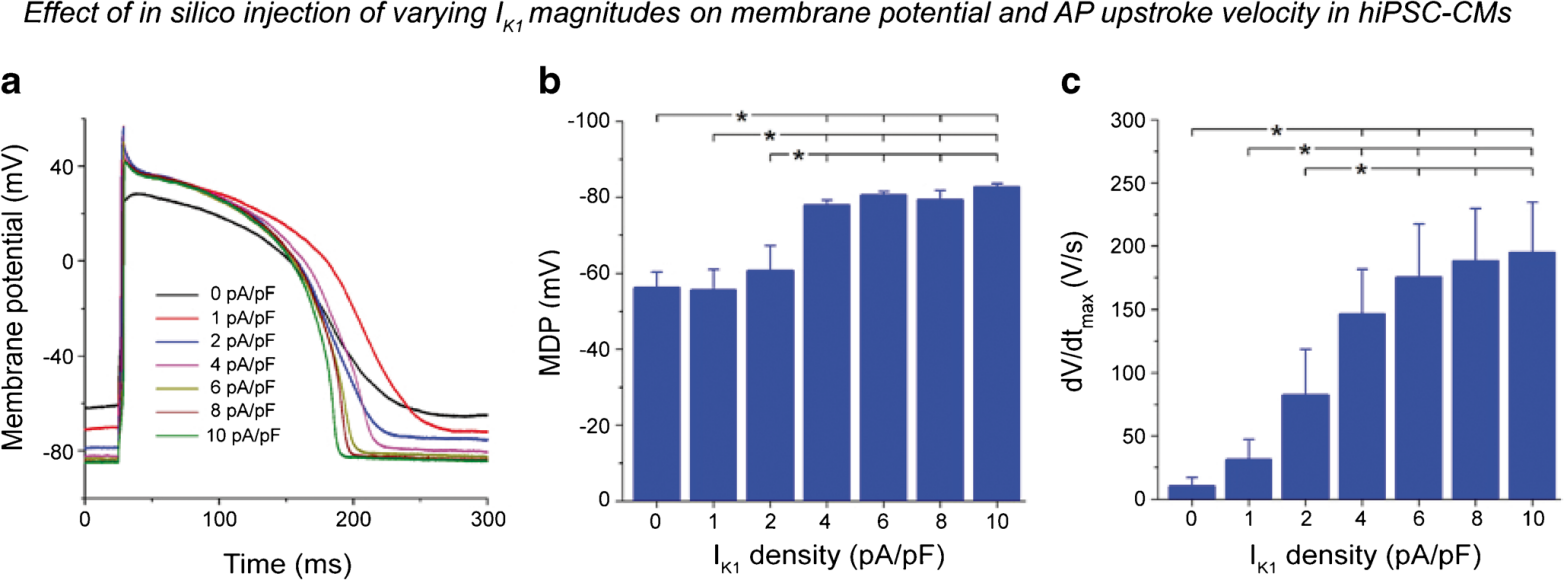
Effect of *I*_K1_ injection on maximal diastolic potential (*MDP*) and maximal upstroke velocity (*dV*/*dt*_*max*_) in hiPSC-CMs. **a** Representative example of action potential traces recorded from hiPSC-CMs upon injection of increasing magnitudes of simulated *I*_K1_ using dynamic clamp. Impact of *I*_K1_ injection at different amplitudes on MDP and dV/dt_max_ is shown in panels **b** and **c**, respectively (*p < 0.05). Figure reproduced from [68]

All techniques described above can be employed for pharmacological studies in hiPSC-CMs research, keeping in mind the advantages and disadvantages of the various approaches. One of the disadvantages is that hiPSC-CMs clusters and monolayers may contain non-cardiomyocytes which influence electrophysiological properties [99]. However, these days, various methods exist to highly purify the population of hiPSC-CMs, including the puromycin and blasticidin selection method [30, 99], lactate treatment [100], and fluorescence-based cell sorting [101]. Many research laboratories are applying approaches aimed at enhancing the structural and functional maturation of hiPSC-CMs. For example, increasing time in culture, medium additives, electrical stimulation, mechanical stretch and/or load, use of polymers, and 3D culture have been shown to improve sarcomeric organization, intracellular calcium handling, contractility, and electrophysiological parameters including MDP and upstroke velocity (see [15, 16, 20, 21]). Furthermore, some limitations are inherent to the lack of *I*_K1_ in hiPSC-CMs resulting in spontaneous activity, depolarized membrane potentials, and difficulties in pacing the cells at the required frequency. To overcome this, a number of groups have employed artificial enhancement of *I*_K1_ density through either viral over-expression of Kir2.1, the channel conducting *I*_K1_ [102, 103], or by injection of an in silico *I*_K1_ with kinetics of Kir2.1 [68, 104] (Fig. 3a–c). Over-expression of Kir2.1 in hESC-CMs [102] abolished cell automaticity, rendering AP characteristics similar to those of adult CMs. Nevertheless, these cells continued to exhibit immature Ca^2+^ handling properties and reduced expression of contractile proteins was reported [102]. In contrast, forced Kir2.1 expression in hiPSC-CMs improved both AP and Ca^2+^ transient properties [103]. This approach may be particularly useful for MEAs and voltage-sensitive fluorescence measurements, although variability in Kir2.1 over-expression levels may enhance heterogeneity. The second approach entails increasing *I*_K1_ through in silico injection of a current with similar properties using the dynamic clamp technique [105]. Bett and colleagues were the first to employ this “electronic expression” of *I*_K1_ in hiPSC-CMs experiments [104]. More recently, our group investigated the in silico injection of varying magnitudes of *I*_K1_ in hiPSC-CMs at physiological temperatures [68] (Fig. 3a–c). In both studies, enhanced *I*_K1_ induced a more physiological and stable RMP, a ventricular-like AP morphology, and increased AP upstroke velocity [68, 104]. Hence, in silico *I*_K1_ injection constitutes a robust tool for improved AP measurements in hiPSC-CMs.

## Pharmacological Studies for Inherited Arrhythmia Syndromes Using hiPSC-CMs

Research into the genetic defects underlying inherited arrhythmia syndromes has increased knowledge on the underlying pro-arrhythmic mechanisms and triggering events. In some cases, this has provided insight into optimal therapeutic strategies for preventing the occurrence of arrhythmias and/or their triggering events [1, 4]. However, for many syndromes, clinical management is still hindered by insufficient knowledge on mutation- and/or patient-specific disease expressivity and treatment efficacy, necessitating development of individualized therapeutic strategies. In vitro and animal models have been used to develop and screen novel pharmacological treatments for inherited arrhythmia disorders. However, these models are not always predictive of drug efficacy in patients, potentially leading to compounds being inappropriately rejected or further developed without success. In this respect, hiPSC-CMs may offer an attractive alternative. Several studies have already shown that hiPSC-CMs can recapitulate the disease phenotype of numerous inherited arrhythmic disorders in vitro (for review, see [29]). The next crucial step involves investigating whether hiPSC-CMs are valid models of cardiac arrhythmia that respond appropriately to clinically relevant pharmacology.

### Long QT Syndrome

Long QT syndrome (LQTS) is an inherited cardiac disorder characterized by QT interval prolongation and increased risk for sudden death due to ventricular tachyarrhythmias, in particular torsades de pointes. At cellular level, LQTS is characterized by AP prolongation and increased incidence of EADs, which can reach the threshold and trigger ventricular arrhythmias. LQTS has been linked to mutations in 15 genes. Long QT syndrome type 1 (LQT1) is the most common form of LQTS, accounting for ≈35% of cases and is associated with loss-of-function mutations in the *KCNQ1* gene, which encodes for the α-subunit of the channel conducting *I*_Ks_. Long QT syndrome type 2 (LQT2), accounting for 30% of cases, arises from loss-of-function mutations in *KNCH2* (also known as hERG), encoding *I*_Kr_. Gain-of-function mutations in *SCN5A*, the gene encoding for the α-subunit of the cardiac sodium channel Na_v_1.5, are linked to LQT3, which accounts for ≈10% of cases. Overall, these three genes account for ≈90% of genotype-positive LQTS patients [1]. LQT1 responds well to beta-blockade, but for LQT2 and LQT3, development of additional therapeutic strategies may improve patient care.

#### Long QT Syndrome Type 1

The first report showing that hiPSCs could model a cardiac channelopathy was the study of Moretti et al. [26], where hiPSC-CMs from a LQT1 patient (carrying the *KCNQ1*-R190Q mutation) recapitulated the electrical phenotype of the disease; patch clamp analysis revealed AP prolongation and decreased *I*_Ks_ in LQT1 hiPSC-CMs as compared to control hiPSC-CMs. Moreover, β-adrenergic stimulation with isoprotenerol exacerbated AP prolongation and facilitated development of EADs in spontaneously beating LQT1 hiPSC-CMs; pre-treatment with propranolol blunted these isoproterenol-induced effects [26]. These observations are in line with the fact that fatal arrhythmias in LQT1 patients are typically precipitated by increased sympathetic tone and are prevented by beta-blocker therapy. In another study modeling LQT1 [61], the *I*_Ks_ blocker chromanol 293B significantly prolonged FPD in control cells but not in LQT1 hiPSC-CMs, suggesting *I*_Ks_ dysfunction. Administration of the *I*_Kr_ blocker E-4031 prolonged FPD in both control and LQT1 hiPSC-CMs but induced frequent severe arrhythmia only in LQT1 hiPSC-CMs [61], indicating reduced repolarization reserve in the latter. Moreover, isoproterenol induced EADs and pro-arrhythmia only in LQT1 hiPSC-CMs, and these were prevented by the beta-blocker propranolol [61]. It has to be noted, however, that in this study, only MEAs were performed and no single-cell AP measurements. In their study, Ma et al. showed that the ML277 compound, a selective *I*_Ks_ activator recently identified, was able to (partly) reverse the decreased *I*_Ks_ and AP prolongation (measured at a relatively depolarized MDP of around −60 mV) in hiPSC-CMs from an LQT1 patient with a deletion mutation in *KCNQ1* [62]. While LQT1 is transmitted in an autosomal dominant manner, homozygous or compound heterozygous mutations in *KCNQ1* or the β-subunit *KCNE1* cause the Jervell and Lange-Nielsen syndrome (JLNS), which has a autosomal recessive pattern of inheritance and is associated with severe life-threatening cardiac arrhythmias, a high risk of sudden death due to ventricular tachycardia, and congenital bilateral deafness [1, 106]. In the study of Zhang et al. [12], hiPSC-CMs from a JNLS patient displayed increased AP duration which was further exacerbated by noradrenaline; this effect was partially reversed by treatment with the β-blocker propranolol. Furthermore, JNLS hiPSC-CMs were more susceptible to arrhythmias when exposed to cisapride, a gastrointestinal drug with known pro-arrhythmic effects through blockade of *I*_Kr_. While beta-blockade is only partially effective in most JNLS patients, in their study, Zhang and colleagues did observe beneficial effects of propranolol on AP duration [12].

#### Long QT Syndrome Type 2

**Fig. 4:**
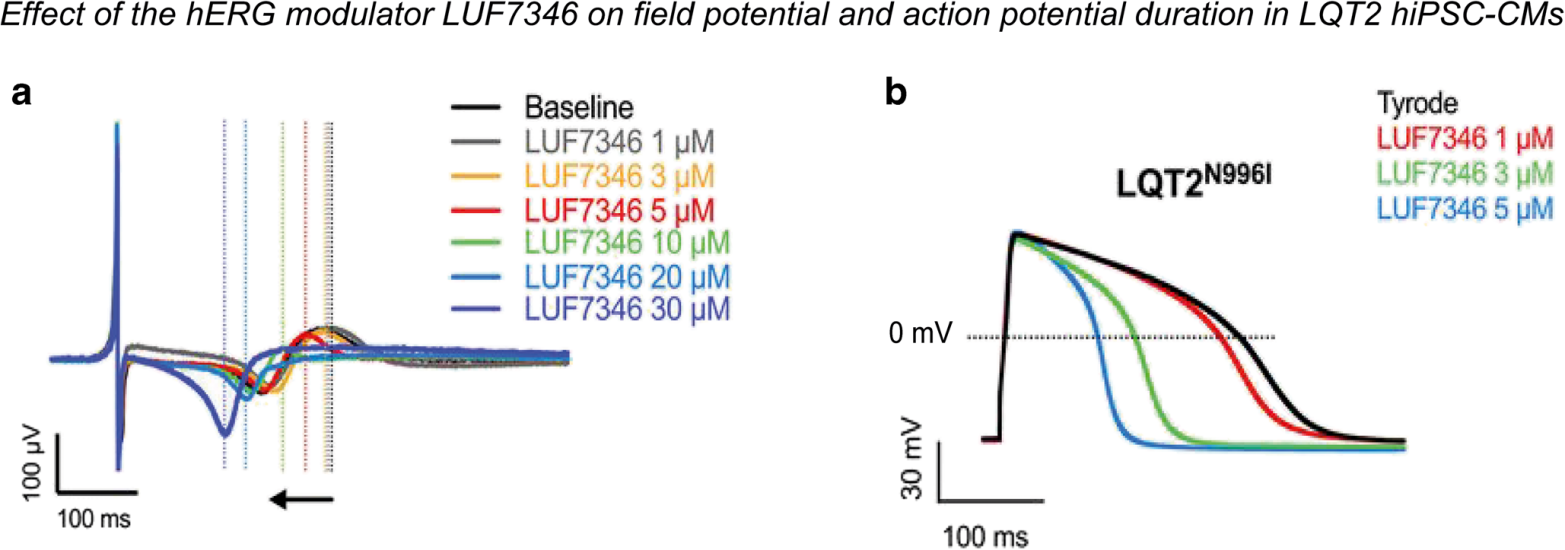
Effect of the hERG allosteric modulator LUF7346 on field potential *(FP)* and action potential (*AP*) duration in LQT2 hiPSC-CMs. **a** Representative FP traces showing the effect of increasing concentrations of LUF7346 on FP duration measured in LQT2 hiPSC-CMs carrying the N996I mutation (LQT2^N996I^). **b** Representative AP traces from LQT2^N996I^ hiPSC-CMs under baseline conditions (Tyrode) and after application of increasing concentrations of LUF7346 (color-code panels are shown in the insets). LUF7346 reduced FP and AP duration in LQT2^N996I^ hiPSC-CMs. Figure modified from [14]

The first report showing the capability of hiPSC-CMs to recapitulate the clinical phenotype of LQT2 was the study of Itzhaki and colleagues, who observed decreased *I*_Kr_, AP prolongation, and marked pro-arrhythmic features (i.e., EADs and triggered activity) in hiPSC-CMs derived from LQT2 patient carrying the *KCNH2*-A614V mutation [55]. Pinacidil, a K_ATP_-channel opener, and nifedipine, an L-type Ca^2+^ channel blocker, caused a significant shortening of both APD and corrected FPD; importantly, pinacidil and nifedipine application completely abolished all EADs and triggered arrhythmias in LQT2 hiPSC-CMs [55]. The potential anti-arrhythmic efficacy of the late Na^+^ current blocker ranolazine was also evaluated. Interestingly, ranolazine application did not significantly alter APD or cFPD, likely due to its nonspecific blocking effect on different ion channels. Nevertheless, ranolazine suppressed triggered activity and EADs in LQT2 hiPSC-CMs [55]. However, the APs in this study showed a depolarized MDP of around −55 mV and a low AP upstroke velocity, indicating relatively immature cells. Furthermore, information on (changes in) MDP was not provided in the drug studies. In the study of Matsa et al., the effect of several compounds was tested in control and LQT2 hiPSC-CMs (*KCNH2*-A561T) [59]. Exposure to E-4031 provoked AP/FPD prolongation in control and LQT2 hiPSC-CMs, with EADs observed only in the mutant cells. In contrast to control CMs, LQT2 hiPSC-CMs also developed EADs when challenged with the β-adrenoreceptor agonist isoprenaline. This effect was reversed by the β-blockers propranolol and nadolol; the latter was also successfully employed as therapy in the patient [59]. In the same study, two experimental potassium channel enhancers were also tested: nicorandil, an *I*_KATP_ channel opener, and PD-118057, an *I*_Kr_ channel enhancer. In LQT2 hiPSC-CMs, both nicorandil and PD-118057 shortened APD by ~18%, with nicorandil also abolishing spontaneously occurring EADs [59]. AP duration was shortened by a further 29.5% (to 58.1%) when combining nicorandil and PD-118057. However, administration of these compounds in combination with isoprenaline induced EADs, suggesting that LQT2 patients treated with potassium channel openers could still be at risk of developing cardiac arrhythmias in the setting of β-adrenoreceptor activation, including exercise. Since these pro-arrhythmic features were prevented by nadolol and propranolol, a treatment strategy combining potassium channel openers with β-blockers was proposed by the authors [59]. It has to be noted, however, that in this particular study, spontaneously beating hiPSC-CMs were used for AP measurements, and no data on MDP was provided. In another study, Lahti et al. [54] tested several compounds with known QT prolongation effects in *KCNH2*-R176W hiPSC-CMs using MEAs. The hERG blocker E-4031 induced EADs in control hiPSC-CMs and even more frequently in LQT2 hiPSC-CMs. In contrast, application of sotalol, an anti-arrhythmic drug with both β-blocker and class III activity, elicited EADs only in LQT2 hiPSC-CMs. No increased arrhythmogenicity was observed with erythromycin or cisapride in either control or LQT2 hiPSC-CMs [54]. Of note, in this study, a decreased *I*_Kr_ was found in hiPSC-CMs obtained from an LQT2 patient carrying this particular mutation (R176W), in line with the clinical phenotype. In contrast, when the same mutation was co-expressed with wild type channels in HEK293 cells, no reduction in *I*_Kr_ was observed [107], underlining the relevance of hiPSC-CMs as disease models. Spencer et al. furthermore showed that the increased AP duration observed in hiPSC-CMs from an LQT2 patient carrying the *KCNH2*-A422T mutation was abbreviated by exposure to the L-type Ca^2+^ channel blocker nifedipine [78]. In addition to assessing the effects of established therapeutic approaches, hiPSC-CMs may also be used for investigating novel compounds. In their study, Sala et al. [14] made use of a series of isogenically matched, diseased, and genetically engineered hiPSC-CMs from patients to test a novel allosteric hERG modulator for treating congenital LQTS, drug-induced LQTS, or a combination of the two. By slowing *I*_Kr_ deactivation and positively shifting its inactivation, the small molecule LUF7346 effectively rescued all of these conditions, demonstrating in a human system that this approach may be useful to treat inherited and drug-induced LQTS [14] (Fig. 4). Similarly, Mehta et al. demonstrated reversal of the LQT2 phenotype (including increased *I*_Kr_, AP shortening at stable but relatively depolarized RMP, and reduced arrhythmogenic events) in *KCNH2*-C1682T hiPSC-CMs by the small molecule *N*-[*N*-(*N*-acetyl-l-leucyl)-l-leucyl]-l-norleucine (ALLN) [56]. This compound was shown to act through restoration of membrane trafficking of hERG, comprising a potential novel therapeutic approach [56]. Taken together, these studies (although in some cases potentially involving relatively immature cells) demonstrate the potential applicability of hiPSC-CMs for pharmacological studies in LQT2.

#### Long QT Syndrome Type 3

**Fig. 5:**
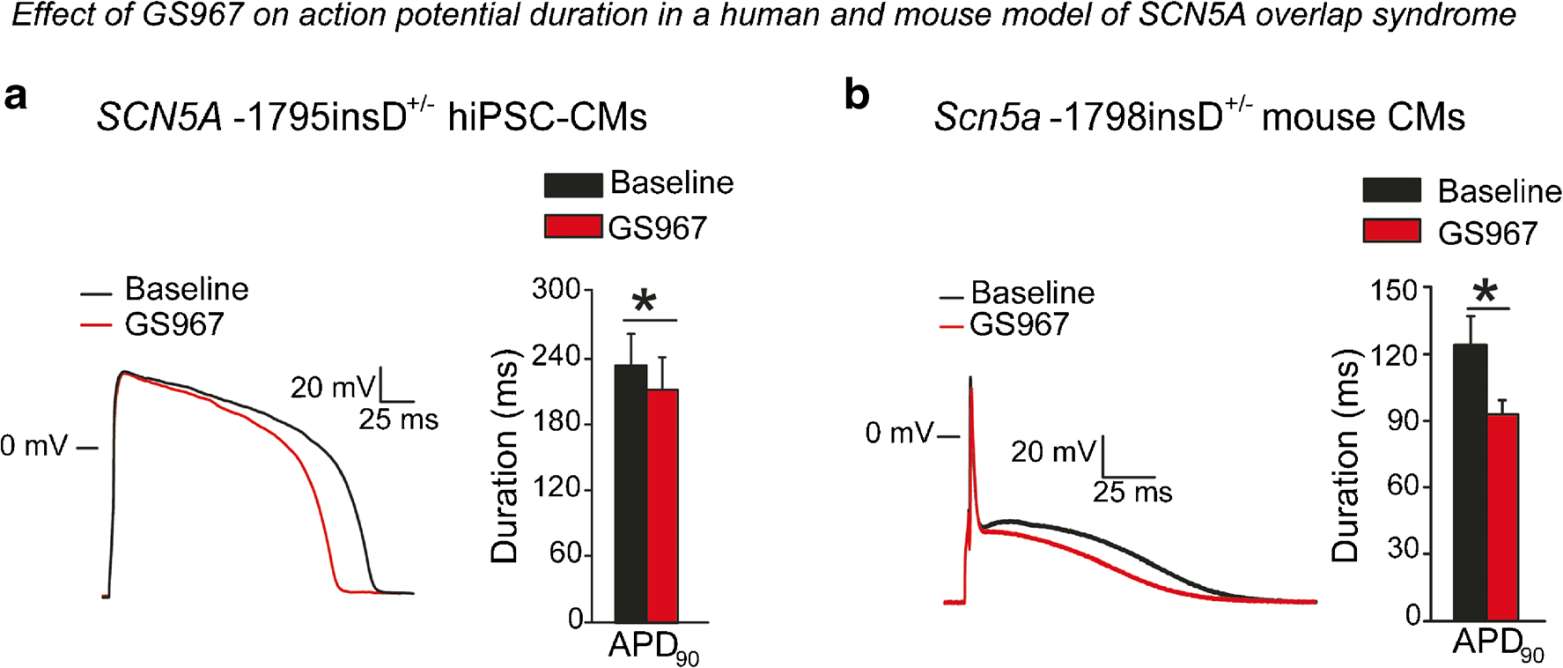
Comparable effect of the late sodium current blocker GS967 in *SCN5A*-1795insD^+/−^ hiPSC-CMs and *Scn5a*-1798insD^+/−^ murine cardiomyocytes. **a**, **b** Representative action potential (*AP*) traces (*left panel*) and average data for AP duration at 90% repolarization (APD_90_, *right panel*) in *SCN5A*-1795insD^+/−^ hiPSC-CMs (**a**) and in *Scn5a*-1798insD^+/−^ cardiomyocytes (**b**) before and after application of GS967. GS967 was able to reduce AP duration in both models. *p < 0.05. Reproduced from [37], with permission

In LQT3, *I*_Na_ inactivation is compromised or channel reopening occurs, leading to *I*_NaL_ which persists throughout the entire AP and ultimately causes AP prolongation and pro-arrhythmia. Some drugs are known to preferentially inhibit this *I*_NaL_, including mexiletine and ranolazine. In hiPSC-CMs from a patient with a de novo LQT3 mutation (*SCN5A-*F1473C) and a polymorphism (K897T) in *KCNH2*, analysis of the biophysical properties of *I*_Na_ and *I*_Kr_ demonstrated that increased *I*_NaL_ was responsible for the pro-arrhythmic phenotype and that the latter was not influenced by the *KCNH2* polymorphism [40]. In the hiPSC-CMs of the patient, increasing the stimulation rate resulted in a 50% decrease in *I*_NaL_; when mexiletine was added, *I*_NaL_ was further reduced. This improved *I*_NaL_ block at the higher stimulation rate was associated with only a modest reduction in peak *I*_Na_, ensuring minimal detrimental impact on conduction. Addition of a second *I*_Na_ blocker, flecainide, did not induce a significant further increase in *I*_NaL_ block at higher stimulation frequency but further reduced peak *I*_Na._ Consistent with these in vitro findings, the most effective arrhythmia control in the patient was achieved by increasing atrial pacing via ICD and by mexiletine treatment [40]. Importantly, while previous studies in heterologous expression systems had reported differing effects of *KCNH2*-K897T on channel function, in hiPSC-CMs, this variant did not impact on anti-arrhythmic drug efficacy. In another study, mexiletine was found to shorten APD and FPD in LQT3 hiPSC-CMs and antagonized EADs in a dose-dependent manner [108]. Moreover, the *I*_NaL_ blockers phenytoin and ranolazine also reduced APDs/FPDs in the same model (*SCN5A*-R1644H), with no effect on control hiPSC-CMs. These findings were in agreement with the pharmacological response profile of the underlying patient carrying the mutation and of other patients from the same family [108]. Similarly, Ma et al. showed that mexiletine reduced *I*_NaL_ and APD in *SCN5A*-V1763M hiPSC-CMs, with little effect on control cells [35]. We recently investigated hiPSC-CMs from a patient carrying the *SCN5A*-1795insD mutation associated with both a gain (LQT3) and a loss (Brugada syndrome, conduction disease) of Na^+^ channel function. The selective *I*_NaL_ blocker GS967 was able to reduce the prolonged AP duration (measured with in silico injection of *I*_K1_) induced by the increased *I*_NaL_ associated with the mutation in these hiPSC-CMs [37] (Fig. 5a). Despite the pre-existent reduced peak *I*_Na_ consequent to the mutation, GS967 only caused a minimal reduction in AP upstroke velocity. Importantly, hiPSC-CMs displayed a similar response to GS967 as adult, freshly isolated CMs from mice harboring the mouse homolog of the mutation *Scn5a*-1798insD [37] (Fig. 5b). In line with the small effect observed on AP upstroke velocity, GS967 did not affect ventricular conduction velocity in isolated wild type or *Scn5a*-1798insD hearts [37]. While these observations strengthen the use of hiPSC-CMs as a valid model for pharmacological studies in inherited sodium channelopathy, complete similarity in results was not observed, since murine *Scn5a*-1798insD CMs displayed EADs and delayed afterdepolarizations (DADs) which were reduced by GS967, but no such pro-arrhythmic features were observed in *SCN5A*-1795insD hiPSC-CMs [37]. This may be due to the relative short APs measured in our hiPSC-CMs, with repolarizing currents suppressing the development of EADs even despite the increased *I*_NaL_.

#### Long QT Syndrome Type 8 (Timothy Syndrome)

A single amino acid substitution (G406R) in *CACNA1C*, the gene encoding for the L-type Ca^2+^ channel, is the cause of Timothy syndrome (long QT syndrome type 8 (LQT8), the most severe variant of LQTS because of its high mortality rate. Besides a marked QT interval prolongation and severe ventricular arrhythmia, LQT8 presents with congenital heart defects, AV block, syndactyly, autism, malignant hypoglycemia, and an abnormal immune system [1]*.* In 2011, Yazawa and colleagues showed that hiPSC-CMs from LQT8 patients recapitulated the electrical phenotype of the disease (prolonged APD, DADs, and altered Ca^2+^ transients) [27]. The experimental drug roscovitine, which alters *I*_CaL_ inactivation, reduced AP duration, restored the irregular Ca^2+^ transient, and decreased the frequency of abnormal depolarizations in LQT8 hiPSC-CMs [27]. However, spontaneously beating cells were used for AP measurements, and therefore a contributory role for alterations in frequency cannot be completely ruled out. Nevertheless, the findings were confirmed in a more recent study employing simultaneous recording of APs and calcium transients in LQT8 hiPSC-CMs using genetically encoded fluorescent indicators [109].

### Brugada Syndrome

Brugada syndrome (BrS) is an inherited disorder characterized by ventricular arrhythmias and sudden cardiac death occurring in otherwise healthy individuals at a relatively young age (*<*40 years), typically during conditions of high vagal tone (i.e., sleep). On ECG analysis, a typical pattern is observed comprising ST-segment elevation in the right-precordial leads V1–V3 [110, 111]. Mechanistically, both depolarization and repolarization abnormalities may be involved, with predominant involvement of the right ventricular outflow tract. In about 20% of BrS patients, a mutation in *SCN5A* has been identified. In general, *SCN5A* mutations associated with BrS are “loss-of-function” mutations, leading to reduced Na^+^ channel availability, either through decreased membrane surface channel expression or through altered channel gating properties [110, 111]. Mutations in other genes have also been sporadically found, and BrS may in fact have a more complex genetic basis than previously thought. In BrS patients at high risk of (recurrent) ventricular tachyarrhythmias, implantation of an ICD should be considered, and pharmacological options are limited. Only few studies so far have recapitulated the BrS phenotype in hiPSC-CMs [31–33], and only one study has tested potential pharmacological interventions. Kosmidis et al. recapitulated the phenotype of two *SCN5A* nonsense mutations (W156X and R1638X) which lead to premature termination of translation and production of truncated proteins [32]. As expected, BrS hiPSC-CMs presented reduced AP upstroke velocity (despite similar MDP) and decreased peak *I*_Na_ as compared to control hiPSC-CMs. In these hiPSC-CMs, the effects of two compounds were investigated, i.e., gentamicin and PTC124, both of which are considered capable of promoting translational readthrough of premature stop codons and hence restoring expression of the full-length, non-truncated protein. While the authors confirmed the readthrough efficacy of the two drugs in HEK293 cells, they did not observe rescue of the electrophysiological phenotype in these BrS hiPSC-CMs [32]. The authors concluded that the effects of the compounds may have been too small to have any functional impact in the setting of the heterozygous BrS mutations.

### Catecholaminergic Polymorphic Ventricular Tachycardia

**Fig. 6:**
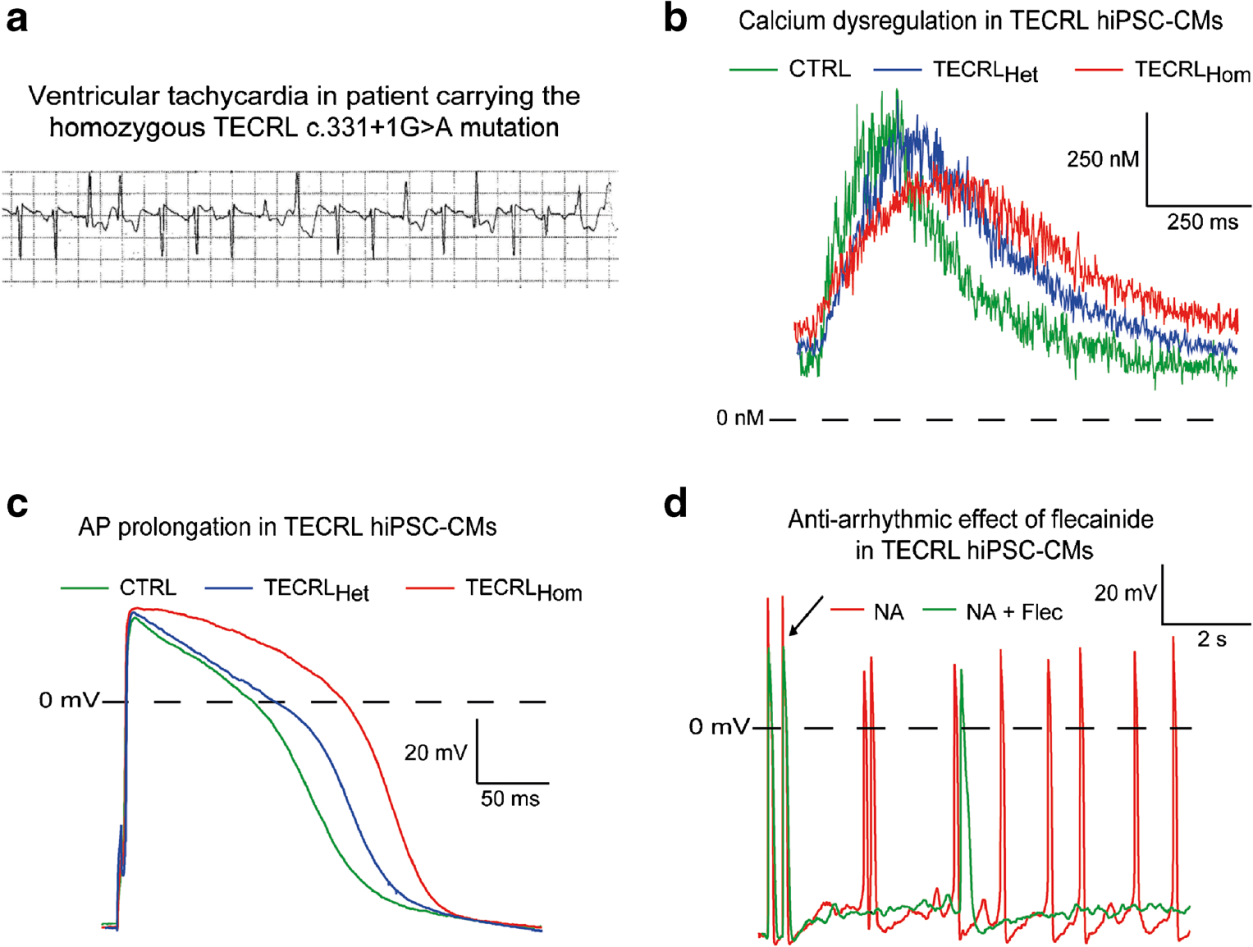
Electrophysiological characteristics of a homozygous mutation in *TECRL*, the gene encoding for the trans-2,3-enoyl-CoA reductase like protein. **a** Example of ventricular tachycardia recorded by the implantable cardioverter defibrillator (*ICD*) in a patient carrying the homozygous splice site mutation c.331+1G>A in *TECRL.* **b**, **c** Representative calcium transient (**b**) and action potential (**c**) traces recorded in control (*CTRL*), heterozygous (*HET*), and homozygous (*HOM*) hiPSC-CMs carrying the *TECRL* mutation c.331+1G>A. The mutation causes a significant increase in diastolic calcium concentrations (**b**) and prolongation of action potential duration (**c**). **d** Addition of 5 μM flecainide (*Flec*) decreased the susceptibility to triggered activity in HOM hiPSC-CMs challenged with noradrenaline (*NA*). Reproduced from [79]

Catecholaminergic polymorphic ventricular tachycardia (CPVT) is an inherited disease characterized by stress and exercise-induced ventricular arrhythmias (in particular, bidirectional VT) in young patients with structurally normal hearts. Autosomal dominant mutations in *RyR2*, the gene encoding for the ryanodine receptor type 2 (CPVT1) or recessive mutations in *CASQ2*, the gene encoding for calsequestrin 2 (CPVT2) are the most common mutations linked to CPVT, resulting in spontaneous Ca^2+^ release from the SR and consequent DADs, triggered activity, and ventricular tachyarrhythmias [112]. The first study modeling CPVT using hiPSCs was performed by Itzaki et al., where CPVT1 hiPSC-CMs carrying the *RYR2*-M4109R mutation showed increased DADs incidence and Ca^2+^ transient irregularities as compared to control hiPSC-CMs, which were further exacerbated by β-adrenergic stimulation [113]. Both flecainide, a sodium channel blocker, and thapsigargin, eliminated DADs in this CPVT1 hiPSC-CMs model [113]. Similar beneficial effects of flecainide were also more recently observed in *RyR*-L3741P hiPSC-CMs, where it improved calcium homeostasis and reduced DADs incidence; clinical efficacy of flecainide was subsequently observed in the mutation carrier [114]. In the study by Jung et al. [115], dantrolene, an inhibitor of sarcoplasmic Ca^2+^ release, which is effective on malignant hyperthermia and is commonly used in the treatment of CPVT patients, restored normal Ca^2+^ handling properties and rescued the arrhythmogenic phenotype in hiPSC-CMs from a CPVT1 patient carrying the S406L mutation in *RyR2*. In a successive study, the antiarrhythmic efficacy of dantrolene during exercise testing was assessed in six patients carrying various *RyR2* mutations [116]. Dantrolene reduced the number of premature ventricular complexes (PVCs) on average by 74% in four patients with N-terminal mutations or mutations in the cytosolic region of the RyR2 protein, while it had no effect in two patients with mutations in or near the transmembrane domain. hiPSC-CMs from the patients replicated these individual drug responses. In hiPSC-CMs with *RyR2* mutations in the N-terminal or cytosolic region, dantrolene abolished the majority of Ca^2+^ transient abnormalities, while it had minimal effect on Ca^2+^ transients in CMs carrying mutations in or near the transmembrane domain [116]. In another hiPSC-CMs model of CPVT1 (*RyR2*-G2311A), the CamKII inhibitor KN-93 abolished isoprotenerol-induced DADs, thereby rescuing the arrhythmogenic phenotype [117]. Of note, results obtained in hiPSC-CMs were in agreement with those previously acquired in a *RyR2* knock-in CPVT mouse model [117]. More recently, Sasaki et al. studied *RyR2*-I4587V hiPSC-CMs and showed that the compound S107, a 1,4-benzothiazepine derivate that stabilizes the closed state of RyR channels (thereby preventing Ca^2+^ leak) by strengthening the interaction between RyR and calstabin, decreased the incidence of isoproterenol-induced DADs [118]. Apart from CPVT1, two recent studies have also investigated hiPSC-CMs model of CPVT2. Lodola et al. observed isoproterenol-induced DADs and, less frequently, triggered activity in hiPSC-CMs carrying the p.G112+5X mutation in the *CASQ2* gene [119]. Over-expression of wild type human *CASQ2* by adeno-associated virus-mediated delivery restored calcium dysregulation and significantly reduced DADs incidence [119]. More recently, Haron-Khun and colleagues investigated the potential involvement of SK4 calcium-activated potassium channels in CPVT2 hiPSC-CMs and mice. The selective SK4 blocker TRAM-34 significantly reduced isoproterenol-induced DADs in *CASQ2*-D307H hiPSC-CMs and freshly isolated, adult ventricular CMs from mice carrying the same mutation [120]. Moreover, TRAM-34 also demonstrated in vivo anti-arrhythmic effects in *Casq2*-D307H knock-in mice during rest and after exercise [120]. Finally, homozygous mutations in *TECRL*, the gene encoding for the trans-2,3-enoyl-CoA reductase like protein, are associated with inherited arrhythmias characterized by clinical features of both LQTS and CPVT [79]. hiPSC-CMs generated from a patient carrying the homozygous splice site mutation c.331+1G>A (Fig. 6a) in *TECRL* (TECRL_HOM_ hiPSC-CMs) revealed increased diastolic Ca^2+^ concentrations, smaller Ca^2+^ transient amplitudes, slower Ca^2+^ rise and decay, as well as prolonged APD compared to hiPSC-CMs generated from his heterozygous but clinically asymptomatic father (TECRL_HET_), and a healthy individual (control, CTRL) [79] (Fig. 6b, c). Increased triggered activity based on the occurrence of delayed afterdepolarizations observed in TECRL_HOM_ hiPSC-CMs following exposure to noradrenaline was reduced by treatment with flecainide [79] (Fig. 6d). Overall, these studies underline the validity of hiPSC-CMs as CPVT disease model and indicate its potential for identifying (novel) therapeutic strategies.

## Conclusions and Perspectives

Despite their potential limitations due to their intrinsic differences compared to adult CMs, hiPSC-CMs are increasingly recognized as valid disease models and tools for pharmacological research. They represent a platform with potential for investigating the molecular pharmacology of ion channel mutations expressed in more complex genetic backgrounds and can provide unique insight into therapeutic approaches for mutation- and/or individual-specific disease management. Studies employing hiPSC-CMs obtained from patients with inherited arrhythmia syndromes have demonstrated clear similarities between in vitro effects of pharmacological interventions and their reported clinical efficacy. This is further confirmed by our recent work demonstrating good correlation between effects of a late *I*_Na_ inhibitor in both hiPSC-CMs and mouse CMs carrying the same *SCN5A* mutation and similar observations by others in CPVT1 mice and hiPSC-CMs [37, 117]. However, the available data on the validity of hiPSC-CMs as models of inherited arrhythmia syndromes is still limited, and effects of pharmacological interventions may vary depending on the gene, the mutation, the individual, as well as technical factors related to hiPSC-CMs use. The availability of novel techniques to genetically modify hiPSC-CMs (including CRISPR-Cas9) will likely accelerate the number of mutations to be studied in the near future and facilitate investigations into mutation-specific therapy. hiPSC-CMs may also be employed to explore other non-pharmacological, gene-based therapeutics, such as allele-specific RNA interference aimed at specifically silencing expression of the allele containing the mutation, without interfering with the normal, non-mutated mRNA [121]. Moreover, the application of automated patch clamp in hiPSC-CMs disease models will facilitate high-throughput drug screens aimed at identifying novel therapeutic compounds, in addition to cardiac safety screening.

Given the expanding spectrum of inherited disorders associated with cardiac arrhythmias, hiPSC-CMs may also be employed for investigating arrhythmogenic mechanisms and potential therapeutic strategies for diseases such as familial atrial fibrillation, conduction disease, arrhythmogenic (right ventricular) cardiomyopathy, and hypertrophic cardiomyopathy. By studying electrophysiological characteristics of hiPSC-CMs from patients with inherited arrhythmia syndromes without an identified causal mutation, the underlying (pro-arrhythmic) disease mechanisms may be identified [33]. Conversely, correcting an identified putative mutation in patient-derived hiPSC-CMs enables establishment of causality [13, 14]. Moreover, comparing hiPSC-CMs from patients carrying the same genetic defect but displaying varying disease expressivity and/or severity may provide insight into the modulatory role of genetic modifiers. Finally, using CRISPR-Cas9 technology, genetic variants may be introduced in hiPSC-CMs lines and their impact on electrophysiological and potential pro-arrhythmic characteristics investigated [12], either in isolation or in combination with a known disease-causing mutation.

Despite their promising use in this wide range of applications, certain limitations intrinsic to hiPSC-CMs need to be considered and addressed. In particular, the issue of immaturity remains an important matter, and techniques aimed at enhancing maturity are continuously being developed and refined, including exposure to electrical stimulation, application of mechanical strain, and culturing hiPSC-CMs in three-dimensional tissue configuration. In addition, artificial enhancement of *I*_K1_ density facilitates accurate assessment of AP parameters. Improved definition and selection of hiPSC-CMs subtype (i.e., atrial, ventricular, Purkinje, nodal) will furthermore allow investigation of disease mechanisms and therapeutic efficacy in a cell type-specific manner. These developments are expected to further strengthen the validity of hiPSC-CMs as models of inherited arrhythmia syndromes as well as other disease entities and their applicability in (future) development of disease-, mutation-, and patient-specific therapies.
